# Using previously genotyped controls in genome-wide association studies (GWAS): application to the Stroke Genetics Network (SiGN)

**DOI:** 10.3389/fgene.2014.00095

**Published:** 2014-04-29

**Authors:** Braxton D. Mitchell, Myriam Fornage, Patrick F. McArdle, Yu-Ching Cheng, Sara L. Pulit, Quenna Wong, Tushar Dave, Stephen R. Williams, Roderick Corriveau, Katrina Gwinn, Kimberly Doheny, Cathy C. Laurie, Stephen S. Rich, Paul I. W. de Bakker, on behalf of the Stroke Genetics Network (SiGN)

**Affiliations:** ^1^Department of Medicine and Program for Personalized and Genomic Medicine, University of Maryland School of MedicineBaltimore, MD, USA; ^2^Veterans Administration Medical CenterBaltimore, MD, USA; ^3^Department of Medicine, University of Texas Health Science CenterHouston, TX, USA; ^4^Department of Medical Genetics, University Medical Center UtrechtUtrecht, Netherlands; ^5^Department of Biostatistics, University of WashingtonSeattle, WA, USA; ^6^School of Medicine, Center for Public Health Genomics, University of VirginiaCharlottesville, VA, USA; ^7^School of Medicine, Cardiovascular Research Center, University of VirginiaCharlottesville, VA, USA; ^8^National Institute of Neurological Disorders and StrokeBethesda, MD, USA; ^9^Center for Inherited Disease Research, Institute of Genetic Medicine, Johns Hopkins University School of MedicineBaltimore, MD, USA; ^10^Department of Epidemiology, University Medical Center UtrechtUtrecht, Netherlands

**Keywords:** genome-wide association study, case-control study, genetic association study, population stratification, power

## Abstract

Genome-wide association studies (GWAS) are widely applied to identify susceptibility loci for a variety of diseases using genotyping arrays that interrogate known polymorphisms throughout the genome. A particular strength of GWAS is that it is unbiased with respect to specific genomic elements (e.g., coding or regulatory regions of genes), and it has revealed important associations that would have never been suspected based on prior knowledge or assumptions. To date, the discovered SNPs associated with complex human traits tend to have small effect sizes, requiring very large sample sizes to achieve robust statistical power. To address these issues, a number of efficient strategies have emerged for conducting GWAS, including combining study results across multiple studies using meta-analysis, collecting cases through electronic health records, and using samples collected from other studies as controls that have already been genotyped and made publicly available (e.g., through deposition of de-identified data into dbGaP or EGA). In certain scenarios, it may be attractive to use already genotyped controls and divert resources to standardized collection, phenotyping, and genotyping of cases only. This strategy, however, requires that careful attention be paid to the choice of “public controls” and to the comparability of genetic data between cases and the public controls to ensure that any allele frequency differences observed between groups is attributable to locus-specific effects rather than to a systematic bias due to poor matching (population stratification) or differential genotype calling (batch effects). The goal of this paper is to describe some of the potential pitfalls in using previously genotyped control data. We focus on considerations related to the choice of control groups, the use of different genotyping platforms, and approaches to deal with population stratification when cases and controls are genotyped across different platforms.

## Introduction

Genome-wide association studies (GWAS) have been widely used in recent years as a tool for identifying susceptibility loci for a number of complex human traits and, in particular, multifactorial diseases. Indeed, the NHGRI-maintained Catalog of Published Genome-Wide Association Studies includes 1788 publications and 12,329 SNP (single nucleotide polymorphism)-trait associations as of 1/10/2013 (http://www.genome.gov/gwastudies/) (Hindorff et al., 2009). With few exceptions, the associated loci have small effect sizes, and large sample sizes were required to detect them.

One popular approach to increase sample size and power for GWAS has been to combine information (either individual-level data or summary statistics) across multiple studies through meta-analysis (Panagiotou et al., 2013). As more and more genotype data are being made publicly available through various databases such as the database of Genotypes and Phenotypes (dbGaP) or the European Genome-phenome Archive (EGA), an alternative approach to increase the statistical power of a study at no extra cost is to devote available clinical and genotyping resources almost entirely to cases and use publicly available data from already genotyped samples as controls. Using available controls may be particularly attractive for registry-based studies from which a large number of cases can be rapidly identified. While this strategy has the obvious benefit of allocating scarce resources toward genotyping a larger number of cases, it can also introduce potential bias into the experimental design leading to spurious associations if not applied carefully. For example, control populations must be comparable to cases in terms of ancestry so that any allele frequency differences observed between cases and controls can be attributed to disease susceptibility loci and not just to differences in ancestral background between the two populations (i.e., population stratification). Ascertainment of the population to be used as controls is also critical. For example, a study of low-density lipoprotein (LDL) concentration could recruit participants at high LDL (a risk factor for stroke) as well as low LDL (not a risk factor); thus, inclusion of all participants as controls could introduce a different bias in the comparison with cases. Equally important is the requirement that genotyping quality and allele calls be comparable between the two groups. Even minor differences in genotype calling, possibly attributable to a laboratory or technician bias, may translate into subtle but systematic differences in allele frequencies between cases and controls that can result in false positive associations.

The goal of this paper is to describe some of the potential pitfalls in using previously genotyped control data. To provide context for these discussions we present as an example the Stroke Genetics Network (SiGN), an international collaboration initiated to carry out a GWAS of ischemic stroke and stroke subtypes that utilizes site-collected cases and already genotyped controls for nearly all sites. We focus on considerations related to the choice of control groups, the use of different genotyping platforms, and approaches to deal with population stratification when cases and controls are genotyped on different platforms.

## Overview of the stroke genetics network (SiGN)

The SiGN was initiated in 2009 to carry out a GWAS of ischemic stroke and stroke subtypes using previously collected DNA samples from multiple centers throughout the US and Europe. These centers included 19 sites contributing 9789 cases to be genotyped. Because of the well-recognized heterogeneity within ischemic stroke, a key feature of SiGN was its focus on standardizing the assignment of stroke subtypes (presumed etiology) for the purpose of performing subtype-specific association analyses. In order to increase the sample size, the decision was made to channel resources into genotyping as many cases as possible and use publicly available control genotypic data wherever possible. A detailed description of the design of SiGN has been previously published, including collection of stroke cases at each study site and the standardizing procedures for assigning stroke subtype (Meschia et al., 2013).

Briefly, stroke research centers with carefully phenotyped ischemic stroke cases were invited to join SiGN and have their stroke cases genotyped using an existing GWAS array. The three requirements for joining SiGN were (1) that the stroke research center have at least 100 cases with DNA immediately available for genotyping, (2) that participating sites must have informed consent on the participants to permit genotypes to be deposited into dbGaP, and (3) that sufficient imaging and additional clinical information had been collected to allow assignment of stroke subtype by Causative Classification of Stroke (CCS) methodology (Ay et al., 2007). CCS phenotyping was performed under a standardized protocol using a web-based system (Meschia et al., 2013).

As indicated in Table 1, the 19 participating sites contributed a total of 11,033 samples for genotyping. With the exception of two sites (Leuven, Belgium and Krakow, Poland) all sites provided cases only. The decision was made to genotype both cases and controls from Leuven and Krakow because of the difficulty in locating previously genotyped controls from those areas.

**Table 1:** Ischemic stroke cases genotyped as part of the SiGN study and previously genotyped control groups, according to study site^*^.

**Site**	**Location**	**Genotype platform**	**Cases (*n*)**	**Controls (*n*)**
**CASES GENOTYPED THROUGH SiGN (US SITES)**				
GASROS	Boston, USA	Illumina HumanOmni 5M Exome	470	
GCNKSS	Greater Cincinnati region, USA	Illumina HumanOmni 5M Exome	499	
ISGS	Multi-center, USA	Illumina HumanOmni 5M Exome	187	
MCISS	New Jersey, USA	Illumina HumanOmni 5M Exome	630	
MIAMISR	Miami, USA	Illumina HumanOmni 5M Exome	299	
NHS	National sample, USA	Illumina HumanOmni 5M Exome	316	
NOMAS(S)	Manhattan, USA	Illumina HumanOmni 5M Exome	363	
REGARDS	National sample, USA	Illumina HumanOmni 5M Exome	311	
SPS3	Multi-center; USA; Latin America, Spain	Illumina HumanOmni 5M Exome	962	
SWISS	Multi-center, USA	Illumina HumanOmni 5M Exome	271	
WHI	National sample, USA	Illumina HumanOmni 5M Exome	458	
WUSTL	St. Louis, USA	Illumina HumanOmni 5M Exome	455	
**CASES GENOTYPED THROUGH SiGN (INTERNATIONAL SITES)**				
BASICMAR	Barcelona, Spain	Illumina HumanOmni 5M Exome	930	
BRAINS	London, England	Illumina HumanOmni 5M Exome	114	
GRAZ	Graz, Austria	Illumina HumanOmni 5M Exome	639	
KRAKOW	Krakow, Poland	Illumina HumanOmni 5M Exome	952	776
LEUVEN	Leuven, Belgium	Illumina HumanOmni 5M Exome	482	468
LUND	Lund, Sweden	Illumina HumanOmni 5M Exome	651	
SAHLSIS	Gothenburg, Sweden	Illumina HumanOmni 5M Exome	800	
**PREVIOUSLY GENOTYPED CONTROL GROUPS**				
HABC	Multi-center, USA	Illumina 1M-Duo		2802
HRS	Multi-center, USA	Illumina HumanOmni 2.5M		12507
OAI	Multi-center, USA	Illumina HumanOmni 2.5M		4011
ADHD	Barcelona, Spain	Illumina HumanOmni 1M		435
GRAZ	Graz, Austria	Illumina 610		829
INMA	Barcelona, Spain	Illumina HumanOmni 1M		1061
KORA	Southern Germany	Illumina Human 550		820
WTCCC	United Kingdom	Illumina 660		5186

* SiGN cases genotyped at the Center for Inherited Diseases (CIDR) on the Illumina HumanOmni 5M Exome array.

Genotyping of SiGN cases was performed at the Center for Inherited Disease Research (CIDR) in Baltimore, Maryland, using the Illumina HumanOmni 5M Exome genotyping array. This array consists of a total of 4,511,703 variants, including 1,084,398 (24%) “rs” (refSNP) SNPs, 3,178,220 (70%) “kgp” (1000 Genomes) SNPs, 231,910 (5%) “exm” (exome) SNPs, and 17,175 (0.4%) other SNPs.

## Improvement in power in SiGN by preferentially genotyping cases

**Figure 1:**
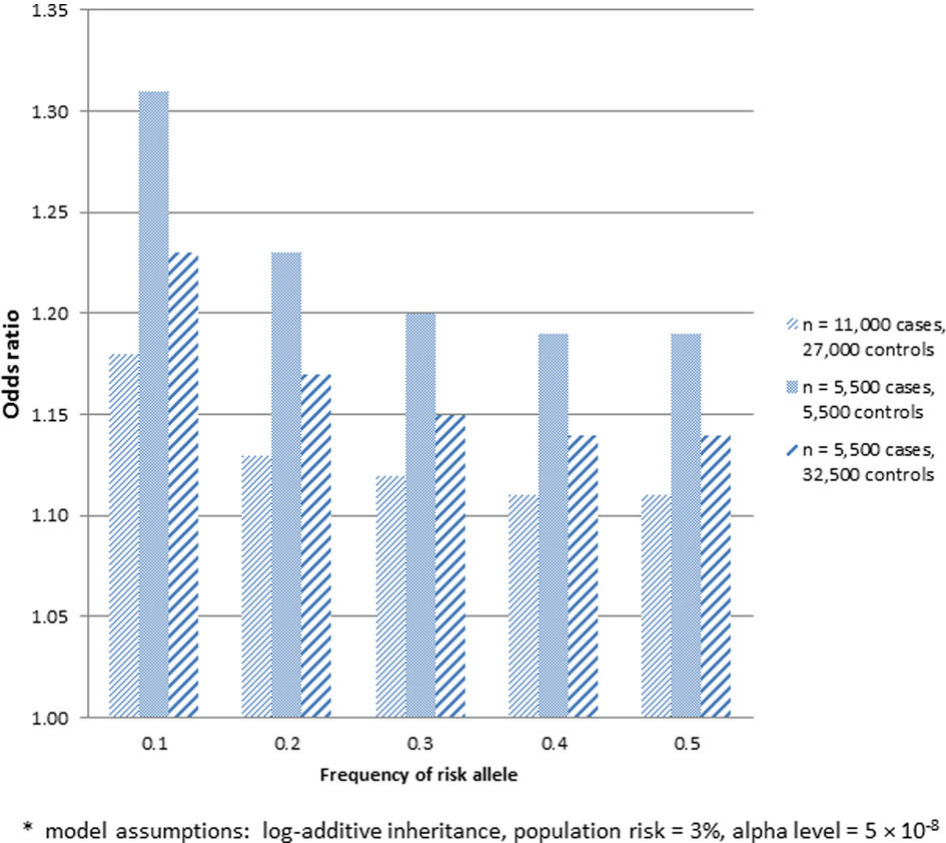
**Minimum odds ratio to detect SNP associations at 80% power for three different sample sets.** (1) 11,000 cases and 27,000 previously genotyped controls; (2) 5500 cases and 5500 controls; and (3) 5500 cases and 27,000 previously genotyped controls^*^.

There may be multiple reasons to consider utilizing already genotyped control groups for a genetic association study. Foremost among these is the increase in sample size of cases for the same genotyping budget to allow detection of variants with smaller effect sizes, assuming a sufficient number of genotyped controls. Within the context of SiGN, we contrasted the power to detect stroke-associated loci using the strategy of genotyping cases only (with already genotyped controls) vs. genotyping a comparable number of cases and controls at each site. The results of these analyses are shown in Figure 1 for a range of allele frequencies and an alpha level of *p* = 5 × 10^−8^. Sample size estimates are guided by the SiGN genotyping budget of ~11,000 subjects. Power is shown for three sets of results: (1) genotyping 11,000 cases and utilizing 27,000 previously genotyped controls (as per SiGN); (2) genotyping 5500 cases and 5500 controls and utilizing no previously genotyped controls; and (3) genotyping 5500 cases and 5500 controls but also utilizing an additional 21,500 previously genotyped controls for a total of 27,000. Shown in Figure 1 are the minimal odds ratios detectable at 80% power at a genome-wide significance alpha level of *p* = 5 × 10^−8^.

As indicated in Figure 1, substantially lower odds ratios can be detected at 80% for sample 1, which includes 11,000 genotyped cases and 27,000 previously genotyped controls, vs. sample 2, which includes only 5500 genotyped cases and 5500 genotyped controls. While much of the gain in power seen in sample 1 comes from the increased number of controls, sample 3 shows that there remains a sizable increase in power in sample 1 that is attributable to genotyping more cases even when the same number of controls is used (e.g., detectable odds ratios of 1.11–1.18 across a range of minor allele frequencies in sample 1 vs. 1.14–1.23 in sample 3). One caveat about applying power calculations to data that includes publicly available controls is that if controls have not been screened for the absence of disease, there is the potential for misclassification and a subsequent loss in power. Potential misclassification was not taken into account in the power calculations above. We further note that such misclassification bias will be more pronounced for common diseases. The power calculations provided also assume equivalent type 1 error rates across the three samples—i.e., no inflation of type 1 error rates introduced by use of publicly available controls.

## Choice of already genotyped control groups

An important consideration in the design of case-control studies is that cases and controls come from comparable underlying populations so that any differences observed between the groups can be attributed to the exposure under study and not to other unmeasured factors that might differ between the groups (i.e., confounding). Compared to other types of epidemiologic studies, however, genetic association studies are well-suited for utilizing already available control groups because of the limited role of confounding in genetic epidemiology studies. When germ-line variation in DNA sequence is the measured exposure of interest, confounding is limited to the presence of population stratification, that is, the ancestral differences between cases and controls. Choosing already genotyped controls from a similar ancestral background as the cases is thus highly important. Fortunately, the high density of coverage of modern SNP platforms makes this a feature that can be empirically investigated from the data themselves without making any assumptions.

The multicenter design of SiGN that included cases with diverse ancestral origins required the inclusion of multiple control groups. In addition to the samples included for GWAS genotyping through CIDR, SiGN also included cases previously genotyped on multiple genotyping arrays and platforms (see Table 1). To reduce variability between cases and controls that could be introduced solely from artifacts related to genotyping platform, the availability of potential controls was limited to those genotyped on a platform believed to be compatible with the Illumina HumanOmni 5M Exome genotyping array used to genotype the cases. This decision limited potential controls groups to sets that had previously been genotyped on a compatible Illumina array and were available to the Network upon request.

Three different multicenter studies were identified for SiGN to serve as controls for the cases from the US sites: The Health and Retirement Study (Juster and Suzman, 1995) (HRS; phs000428.v1.p1), The Osteoarthritis Initiative (Lester, 2008) (OAI; http://www.oai.ucsf.edu/datarelease/About.asp), and HealthABC (Yaffe et al., 2009) (HABC; phs000169.v1.p1). These studies were selected because of their large sizes (12,500, 4000, and 2800, for HRS, OAI, and HABC, respectively), their geographic diversity within the US, and the dense genotyping available on each (Illumina HumanOmni 2.5M array in HRS and OAI and Illumina Human 1M array for HABC). All three studies included substantial representation of European Caucasians and African Americans, while HRS also included substantial numbers of Hispanics.

For each international (non-US) site, studies with previously genotyped controls were identified from the same ancestral background. For two sites (Leuven and Krakow), previously genotyped ancestry-matched control groups could not be identified, and so controls at these sites were genotyped alongside SiGN cases. For other sites (e.g., Barcelona), multiple control groups were identified to allow cases to be matched to suitable controls at a later stage, where we initially included as many genotyped controls as possible to improve power.

## Availability of disease risk factors and other covariates

One drawback of using an already available control group is that that the clinical and covariate data may be limited or even absent altogether. This issue is of particular importance when effect decomposition is of interest, for example, whether a SNP acts through a modifiable risk factors such as smoking, or interacts with such a factor (Vanderweele and Hernan, 2012). Additionally, utilizing properly selected publically available controls can produce unbiased estimates of total genetic effects, even in the presence of gene by environment interactions, but these estimates may not be generalizable to populations with drastically different covariate distributions. If covariate information is missing in the controls, extending research findings to other populations may be limited. This limitation is mitigated in the absence of gene by environment interaction or the low prevalence of the genetic variant.

A second potential drawback of using already available controls is that misclassification bias can result if controls are not “disease free.” In studying an aging-related disease such as stroke, one may want to choose already genotyped controls that are disease-free and older so that genetically susceptible individuals are under-represented in the control pool. To the extent that phenotypic characterization is limited and disease status unknown, the use of publicly available controls may be better suited for studies of rare/uncommon diseases for which the likelihood that controls are affected is small. The prevalence of stroke in the adult population is approximately 3–4% (Go et al., 2014). We note that misclassification bias only reduces power and does not influence type 1 error (false positives).

## Comparability of genotyping platforms between cases and controls

In case-control studies it is critical to obtain measurements from cases and controls in comparable fashion to ensure that any measurement differences between groups are not due to artifacts in measurement procedures. In the case of genetic association studies, spurious differences between cases and controls can occur by virtue of systematic differences in sample processing, genotype assays (choice of genotyping platform), and genotype calling procedures. Potential biases due to different genotyping procedures constitute perhaps the biggest challenge for genetic association studies that utilize previously genotyped controls. This potential source of bias can be minimized by choosing control groups that have been previously genotyped on the same, or a highly compatible, platform as the one used for cases.

As additional quality control, it may be useful to genotype a small number of previously genotyped individuals alongside the cases to evaluate genotype discordance across different platforms. SiGN cases are genotyped using the Illumina HumanOmni 5 M Exome genotyping array, but controls had previously been genotyped on different arrays, primarily the Illumina Omni 1 M and the Illumina Omni 2.5 M. To evaluate genotyping quality between these platforms, DNA from 30 previously genotyped subjects were identified from five of the control populations (HRS, OAI, INMA GRAZ, and LUND) and then re-genotyped at CIDR alongside the cases so that genotype calls from the same sample could be compared across the two arrays. Genotype concordance rates were calculated across each set of 30 samples and all SNPs having one or more discordant genotypes (*n* = 17,401 SNPs) were flagged as potentially problematic and excluded from subsequent imputation and case-control analysis. The effectiveness of this filter can be evaluated empirically by assessing type 1 error rates in association analysis among these SNPs.

## Post-genotyping quality control procedures to enhance genotype comparability between cases and controls

Differential genotyping quality between newly genotyped cases and previously genotyped controls is a primary source of spurious results in GWAS. Thorough quality control analysis of the case and control data sets is therefore critical. The first step is to identify poor quality samples in cases and controls and remove these from further analyses. In SiGN preference was given to control groups genotyped and cleaned by the same labs that processed case genotype data. Genotyping of cases was performed on the Illumina HumanOmni5Exome-4v1 array at CIDR, which also performed initial quality control (QC), including manual review and, as needed, manual re-clustering of SNPs selected as potentially problematic (such as many low minor allele frequency exome SNPs). Additional QC was performed at the University of Washington using methods described by Laurie et al. (2010) in general and by Meschia et al. (2013) specifically for SiGN. Among ~11,000 subjects genotyped, 8 were excluded due to unresolved identity issues (e.g., sex mismatches and unexpected relatedness).

Using the KING-robust method (Manichaikul et al., 2010) to analyze cryptic relatedness revealed that 99% of the subjects are mutually unrelated. We defined a related pair of subjects as connected by a kinship coefficient achieving the lower limit of the 95% prediction interval for second-degree relative pairs (KC > 0.088). Among 4.5 million SNPs assayed, 4.2% were either failed by CIDR or flagged as potentially low quality. Starting with 4.3M non-monomorphic and unique SNPs, 110K were failed by CIDR (for various reasons, including manual review of zCall-flagged SNPs), an additional 60K for missing call rate ≥2%, an additional 5K for 3 or more discordant calls among 343 duplicate pairs, an additional 1.8K for 2 or more Mendelian errors among 24 HapMap trios, and an additional 2.5K for HWE *p*-value <0.0001 (in controls only), resulting in 4.1M SNPs passing QC. The median call rate was 99.9% and the error rate estimated from 343 pairs of sample duplicates was 2 × 10^−5^, indicating very high quality data.

The QC procedures were applied to all SNPs regardless of minor allele frequency, but standard quality metrics have less power to detect problems with rare than with common variants. A post-processing procedure has been proposed for modifying GenomeStudio calls to improve accuracy of genotypes for rare variants (Goldstein et al., 2012). CIDR used a modified version of zCall to flag SNPs with potential problems as those with specific differences in genotype calling between GenomeStudio and zCall. Specifically, the CIDR QC process for low MAF SNPs includes running zCall to identify SNPs where possible heterozygous clusters were missed by GenCall (parameters *T* = 21 and *I* = 0.2). SNPs with 4 or more possible new heterozygotes were manually reviewed and manually re-called (or failed) as needed.

## Assessment of population stratification, imputation, and data analysis strategy

**Figure 2:**
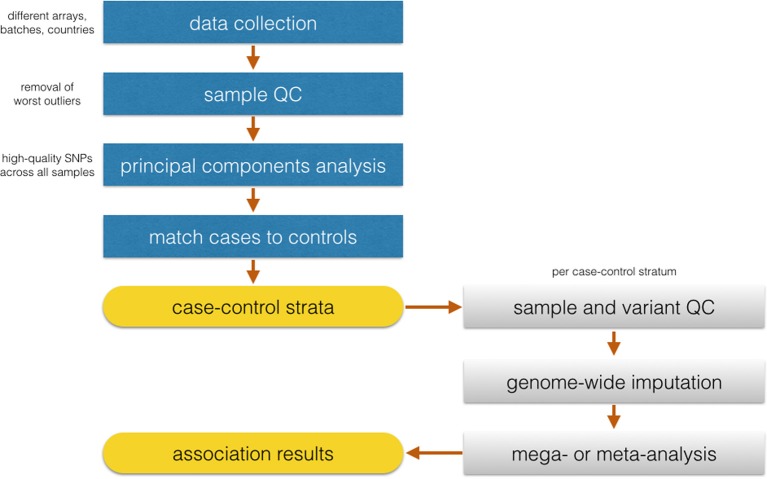
**Flowchart of the proposed analysis by SiGN**.

Identifying matching control groups from the same ancestral background as cases is a necessary step to ensure case-control comparability in GWAS studies. Analysis of population substructure was particularly challenging in SiGN because of the desirability in generating population substrata that were based not only on ancestry but also on array content to minimize the pairing of samples genotyped on very dense arrays (e.g., Illumina HumanOmni 5M Exome) with samples genotyped on relatively sparse (e.g., Illumina 610) arrays. The approach we took in SiGN to accommodate these two competing strategies is summarized in Figure 2. Our first step was to define four array groups (Illumina 610, Illumina 660, Illumina 1M, and Illumina 2.5/5M). Within each array group, we then defined three different continental groupings (Europe, Africa, and Admixed) using principal components analysis (PCA) (Price et al., 2010), projecting onto the HapMap 3 samples. Only “high-quality” SNPs were used for these analyses, defined as those with extremely low missingness (e.g., <0.1%) on all platforms, high frequency (e.g., >20%, as these are easier to genotype than low-frequency SNPs), outside of regions, such as the MHC or lactase (*LCT*) gene, that tend to be highly diverse even across populations of similar ancestry, and LD-pruned at an r^2^ of 0.2.

Once continental groupings were defined within array groups, we then performed a second round of quality control analyses within ancestry by array group strata to remove problematic samples and SNPs, such as those samples or SNPs with high missingness rates or samples with inbreeding coefficients further than 3 SD from the mean of the sample distribution.

With the QCed set of samples, the next task was to combine continental groupings across array groupings to investigate population stratification across the full study sample. To do this, we started with a set of SNPs that were common across all samples and arrays (*n* = 206,476 SNPS) and selected high-quality sites only (as described above). After this SNP selection, the remaining 50–60K SNPs (depending on continental group) were used for PC analysis to check case-control clustering across all groups. Only 10 cases were missing matched controls and were removed from the analysis.

Iterative logistic regression and evaluation of statistical inflation (lambda) (Devlin and Roeder, 1999) will be necessary to recognize the extent of false-positives in the data and remove SNPs showing association to the trait due to systematic genotyping differences. Following identification of discrete case-control strata with well-behaved association statistics, imputation will be performed in continent-specific and array-specific groups. The SiGN analysis plan is for case-control analysis for stroke and its subtypes to be performed separately within each stratum using logistic regression, and then merged across strata using standard meta-analysis procedures.

## Summary

GWAS have been undeniably successful in identifying novel disease susceptibility loci (e.g., Billings and Florez, 2010; Teslovich et al., 2010; Chasman et al., 2012). Nonetheless, results from GWAS have also made clear that very large sample sizes are required to detect trait-associated SNPs that have small effect sizes. Large collections of cases suitable for genetic studies can often be obtained by pooling cases from a variety of sources, such as case reports, registries or large epidemiologic studies or, as demonstrated more recently, through the use of electronic health records (Ritchie et al., 2010). As we describe in this manuscript, there can be immense efficiency achieved in power by devoting genotyping resources to cases and using previously genotyped controls.

Availability of large collections of previously genotyped controls has been greatly facilitated by the decision of NIH that all genotypes for GWAS studies funded by federal dollars be made available for further research. In 2007 the tool dbGaP was introduced to facilitate community-wide access to these data (Mailman et al., 2007). It was this decision, the making available of publicly funded genotyping data, that affords researchers the opportunity to expand further scientific discoveries, as outlined here. Genetic researchers are thus favorably positioned to take advantage of this tremendous resource and are not as beholden to the initial study design as other etiologic research. This benefit does not come without a cost. We have outlined here, and summarized in Table 2, some considerations researchers may wish to consider as they design case-control studies using publicly available data.

**Table 2:** **Considerations when using already genotyped controls**.

**SELECTION OF CONTROLS:**
• When possible, identify control sets that are from similar ethnic ancestry and were genotyped on the same platform as the cases.
• Consider using multiple control groups, especially when cases and controls are genotyped on different platforms and/or when the size of available control groups is small.
• Cross-study duplicates: if possible, re-genotype a small number of previously genotyped controls to allow evaluation of SNP concordance rates across the two platforms.
**POPULATION SUBSTRUCTURE, IMPUTATION, AND ASSOCIATION ANALYSIS:**
• Combine cases and previously genotyped controls together for assessment of population substructure, using a subset of non-imputed markers common to all samples (and after excluding SNPs found to be discordant from analysis of cross-study duplicates).
• Impute genotypes of cases and controls within population substrata.
• For confirmation, it is prudent to replicate observed associations after re-genotyping cases and control samples together.

### Conflict of interest statement

The authors declare that the research was conducted in the absence of any commercial or financial relationships that could be construed as a potential conflict of interest.
